# The copper transporter CTR1 and cisplatin accumulation at the single-cell level by LA-ICP-TOFMS

**DOI:** 10.3389/fmolb.2022.1055356

**Published:** 2022-11-28

**Authors:** Anna Schoeberl, Michael Gutmann, Sarah Theiner, Mario Corte-Rodríguez, Gabriel Braun, Petra Vician, Walter Berger, Gunda Koellensperger

**Affiliations:** ^1^ Institute of Analytical Chemistry, Faculty of Chemistry, University of Vienna, Vienna, Austria; ^2^ Center for Cancer Research and Comprehensive Cancer Center, Medical University of Vienna, Vienna, Austria; ^3^ Department of Physical and Analytical Chemistry, Faculty of Chemistry and Instituto de Investigación Sanitaria del Principado de Asturias (ISPA), University of Oviedo, Oviedo, Spain

**Keywords:** CTR1 copper transporter, laser ablation, cisplatin accumulation, single-cell, cisplatin resistance, ICP-TOFMS

## Abstract

More than a decade ago, studies on cellular cisplatin accumulation *via* active membrane transport established the role of the high affinity copper uptake protein 1 (CTR1) as a main uptake route besides passive diffusion. In this work, CTR1 expression, cisplatin accumulation and intracellular copper concentration was assessed for single cells revisiting the case of CTR1 in the context of acquired cisplatin resistance. The single-cell workflow designed for *in vitro* experiments enabled quantitative imaging at resolutions down to 1 µm by laser ablation-inductively coupled plasma-time-of-flight mass spectrometry (LA-ICP-TOFMS). Cisplatin-sensitive ovarian carcinoma cells A2780 as compared to the cisplatin-resistant subline A2780cis were investigated. Intracellular cisplatin and copper levels were absolutely quantified for thousands of individual cells, while for CTR1, relative differences of total CTR1 versus plasma membrane-bound CTR1 were determined. A markedly decreased intracellular cisplatin concentration accompanied by reduced copper concentrations was observed for single A2780cis cells, along with a distinctly reduced (total) CTR1 level as compared to the parental cell model. Interestingly, a significantly different proportion of plasma membrane-bound versus total CTR1 in untreated A2780 as compared to A2780cis cells was observed. This proportion changed in both models upon cisplatin exposure. Statistical analysis revealed a significant correlation between total and plasma membrane-bound CTR1 expression and cisplatin accumulation at the single-cell level in both A2780 and A2780cis cells. Thus, our study recapitulates the crosstalk of copper homeostasis and cisplatin uptake, and also indicates a complex interplay between subcellular CTR1 localization and cellular cisplatin accumulation as a driver for acquired resistance development.

## Introduction

Cisplatin and its anticancer properties were discovered by Rosenberg et al. (1965) leading to clinical approval as chemotherapeutic drug in 1978. Up to date, it remains one of the most widely administered metal-based therapy for the treatment of various malignant diseases, such as bladder, ovarian, head and neck, lung, and testicular cancer (Galanski, 2006; Dasari and Bernard Tchounwou, 2014). The applicable therapeutic schemes are limited by severe side effects such as dose-limiting nephrotoxicity and therapy failure, based on intrinsic and/or acquired resistance phenotypes (Abada and Howell, 2010; Oun et al., 2018). As one prominent example, cisplatin was the first platinum-based compound approved by the Food and Drug Administration (FDA) for the treatment of one of the most common gynecologic tumors worldwide, ovarian cancer. However, despite potent initial responses, most patients acquire resistance to the drug, leading to therapeutic failure and increased mortality (Song et al., 2022).

Cisplatin resistance development was associated to reduced intracellular drug accumulation and several factors were identified to contribute to the resistance phenotype such as decreased uptake and/or increased efflux, elevated drug inactivation within the cell, enhanced DNA repair and/or tolerance to DNA damage, alterations within the tumor microenvironment, and evasion of the host immune response (Kalayda et al., 2012; Song et al., 2022).

Drug efflux is regulated *via* efflux transporters. Examples for these are the copper-transporting ATPase 1 and 2 (ATP7A and B). The main function of these transporters is the removal of excess Cu from cells, but also the cellular efflux of cisplatin can be regulated. Studies have indicated, that patients suffering from lung and ovarian cancer show a poor response to cisplatin when high levels of ATP7A and/or ATP7B are expressed. In addition, multidrug resistance-associated proteins (MRPs) and members of ATP-binding cassette (ABC) transporters were suggested to mediate cellular resistance to cisplatin (Chen and Chang, 2019).

The main route of cellular cisplatin uptake is passive diffusion, but also active uptake through membrane transporters was described to play a key role. Examples here are the high affinity copper uptake protein 1 and 2 (CTR1 and CTR2, respectively) and the organic cation transporter 2 (OCT2). CTR2 is mainly located in intracellular vesicles. Recent studies suggested that CTR2 can induce a cleavage of CTR1, which leads to reduced uptake of cisplatin, and a high expression of CTR2 was associated with poor survival in ovarian tumor patients. OCT2 is frequently expressed in the kidney and also responsible for cellular transportation of cisplatin. The most described uptake transporter is CTR1 (Chen and Chang, 2019). Several studies related the high affinity copper uptake protein 1 (CTR1) to the uptake of platinum-based chemotherapeutic drugs (Holzer et al., 2006; Chen and Chang, 2019). The human CTR1 (encoded by the *SLC31A1* gene) belongs to the superfamily of membrane spanning transport proteins responsible for dietary copper homeostasis in mammalian cells (Garmann et al., 2008). One of the first indications that parts of the copper (Cu) homeostasis machinery are involved in resistance development to platinum drugs was provided by the observation of cross-resistances of cells against cisplatin and other metals or metalloid-containing agents (Naredi et al., 1994, 1995; Shen et al., 1998). Subsequently, the crucial role of CTR1 in the active transport of platinum drugs was demonstrated by numerous research groups in yeast, mouse and human cells (Ishida et al., 2002; Lin et al., 2002; Holzer and Katano, 2004; Safaei and Howell, 2005; Safaei, 2006). Deletion of the CTR1 gene in yeast and mammalian cells resulted in reduced cisplatin uptake and increased resistance to the metal drug. In a study of Ishida et al. (2002) they could show that *S. cerevisiae*, which were lacking the CTR1 transporter, were resistant to cisplatin resulting in a reduced accumulation of the drug. In addition, similar observations of a drug resistance through lowered uptake were made in mouse embryo fibroblasts lacking one or both CTR1 alleles. Notably, Lin et al. (2002) identified that CTR1 mediated the uptake of the other two worldwide clinically approved Pt drugs, i.e., carboplatin and oxaliplatin, as well as other cisplatin analogues using yeast cells. In addition, markedly elevated Cu and Pt levels were demonstrated in the human ovarian cancer cell line A2780 following a 20-fold overexpression of CTR1 (Holzer and Samimi, 2004). The relevance of Cu import transporters for cellular Pt uptake and sensitivity and the correlation between CTR1 and intracellular Pt levels was further shown in ovarian and cervical cancer cell models sensitive or resistant to cisplatin (Song et al., 2004; Zisowsky et al., 2007). The findings of this study highlighted a robust correlation between distinctly decreased CTR1 expression levels and reduced intracellular Pt concentrations, attenuated DNA platination and an overall decreased platinum sensitivity in the resistant cancer cell sublines (Zisowsky et al., 2007). In addition, it was demonstrated that cisplatin is capable of inducing the degradation of CTR1, leading to a limited drug accumulation (Blair et al., 2010). Another study found, that exposure of cultured human ovarian carcinoma cell lines to cisplatin downregulated CTR1 transporter in a concentration- and time-dependent manner. Incubation of A2780 cells with 1 µmol L^−1^ caused nearly complete loss of all CTR1 immunostaining (Holzer and Katano, 2004). The authors found CTR1 associated with the plasma membrane and with vesicular structures scattered throughout the perinuclear region. In agreement with previous reports, Schneider et al. (2017) was able to highlight a significant reduction in CTR1 expression levels in the ovarian carcinoma cell line A2780 after exposure to cisplatin and carboplatin in combination with the multi-kinase inhibitor Sorafenib. Surprisingly, the treatment did not affect CTR1 expression in the cisplatin-resistant ovarian carcinoma A2780cis cell line (Schneider et al., 2017). In contradiction to those findings, cisplatin induced loss of CTR1 and cisplatin exposure had no remarkable impact on the sub-cellular localization of CTR1 in the A2780 and A2780cis cell models (Kalayda et al., 2012). The clinical relevance of CTR1 expression levels was further demonstrated in ovarian carcinoma patients: elevated expression of CTR1 was associated with enhanced therapeutic responses, while low transporter levels worsened the therapeutic outcome (Lee et al., 2011).

Although several studies demonstrated the importance of the CTR1 transporter for platinum-based anticancer chemotherapy, knowledge about the exact interplay of cisplatin uptake and the abundance of CTR1 in a quantitative manner at the single-cell level is still lacking. Additionally, contradictory observations in the literature concerning the ability of cisplatin itself to downmodulate CTR1 expression need to be clarified at single-cell resolution. Hence, new methods need to be applied to answer these unsolved problems. Our group recently introduced a method using laser ablation-inductively coupled plasma-time-of-flight mass spectrometry (LA-ICP-TOFMS) of single cells, deposited on microscope slides using a cytospin preparation technique, which allows the quantitative assessment of thousands of single cells in an appropriate time regime (Schoeberl et al., 2021). This method uses the potential of newest low dispersion laser ablation setups, which allow the analysis of single cells and (sub)cellular imaging (with spot sizes down to 1 µm or even lower) at pixel acquisition rates of >200 Hz (Malderen et al., 2016; Van Malderen et al., 2020). In combination with a time-of-flight-based ICP-MS instrument (ICP-TOFMS), which enables the quasi-simultaneous detection of all elements of the periodic table (Gundlach-Graham and Günther, 2016; Malderen et al., 2016), and the labeling of cellular markers using metal-tagged antibodies, introduced by mass cytometry (Lou et al., 2007; Bandura et al., 2009; Chang et al., 2017), the total amount of platinum per cell and its relationship with the CTR1 receptor together with trace elements can be investigated. The potential of single-cell LA-ICP-MS was already comprehensively described elsewhere (Theiner et al., 2019, 2020, 2021; Van Acker et al., 2019).

Herein, we employed the ovarian cancer cell line A2780 and its cisplatin-resistant subline A2780cis to measure the cellular amount of CTR1 transporters and metal-drug uptake after exposure to cisplatin. We were able to analyze several thousands of single cells on a quantitative basis in a time regime of minutes. An automated cell segmentation facilitated rapid data evaluation of thousands of cells allowing meaningful statistical analyses.

## Materials and methods

### Chemicals and reagents

Ultrapure water (18.2 MΩ cm, ELGA Water purification system, Purelab Ultra MK2, United Kingdom) and nitric acid (>69%, Rotipuran Supra, Carl Roth, Karlsruhe, Germany) were used for all dilutions for standard preparation. Ultrapure water was additionally used for all dilutions in the labeling procedure. For the production of the gelatin standards, a multi-element stock solution and single element standard solutions, purchased from Labkings (Hilversum, Netherlands), as well as gelatin (from cold water fish skin), obtained from Sigma-Aldrich (Vienna, Austria), were used. A Cell-ID Intercalator-Ir (125 µM), for labeling of the nuclei, and a Maxpar^®^ X8 Antibody Labeling Kit containing ^165^Ho, for the labeling of the CTR1 antibody, were purchased from Fluidigm (San Francisco, CA, United States). A recombinant anti-SLC31A1/CTR1 antibody was obtained from Abcam (Cambridge, United Kingdom). For antigen retrieval, a target retrieval solution (pH 9) from Agilent Technologies (Waldbronn, Germany) was used. Bovine serum albumin (BSA) and tris buffered saline (TBS) were obtained from Sigma-Aldrich (St. Louis, MO, United States). An antibody stabilizer solution was obtained from Candor Bioscience (Wangen im Allgäu, Germany). Amicon Ultra filters with sizes of 3 and 50 kDa were purchased from Merck (Darmstadt, Germany). Tween™ 20 Surfact-Amps™ Detergent Solution (10%) for cell permeabilization was obtained from Thermo Scientific (Waltham, MA, United States).

Sample preparation (except cell culture) and all ICP-MS measurements were carried out in clean room ISO class 8 and 7, respectively. Unless stated otherwise, all cell culture media and reagents were purchased from Sigma-Aldrich (St. Louis, MO, United States) and all plastic dishes, plates and flasks from StarLab (Hamburg, Germany). Cisplatin for cell treatment was synthesized at the Institute of Inorganic Chemistry, University of Vienna, according to literature procedures (Dhara, 1970). Stock solutions of cisplatin were prepared as 55 mM solutions in dimethylformamide (DMF), further diluted with RPMI 1,640 cell culture medium to obtain a working solution of 5 mM with 10% DMF, which was stored at −20°C. TrypLE™ Express with phenol red (Gibco, Fisher Scientific, Roskilde, Denmark) was used for gentle cell detachment from culture plastic following the instructions of the manufacturer.

### ^165^Ho-labeling of the anti-CTR1 antibody

A BSA and azide-free recombinant anti-SLC31A1/CTR1 antibody, reacting with the human CTR1, was purchased from Abcam (#ab240041), and labeled using a Maxpar X8 antibody labeling kit (containing ^165^Ho) according to the MAXPAR manufacturer’s protocol (Han et al., 2018). Briefly, the labeling procedure consisted of three steps, first the polymer was loaded with the lanthanide solution (^165^HoCl), second the antibody was partly reduced using tris(2-carboxyethyl)phosphine (TCEP), and third the antibody was conjugated with the lanthanide-loaded polymer. After conjugation, the antibodies were diluted to a final concentration of 0.5 mg L^−1^ using an antibody stabilizer solution and stored at −20°C. The concentration was measured using a NanoDrop instrument. To verify whether the labeling process was successful, a size exclusion ICP-QQQ-MS measurement was performed using the sulfur signal to visualize the antibody and the ^165^Ho signal to show the presence of the metal tag. An overlay of those two signals represented a successful lanthanide-labeling of the CTR1 antibody.

### Cell models and cell culture conditions

The human ovarian cancer cell line A2780 and the cisplatin-resistant cell line A2780cis, obtained from Sigma-Aldrich were maintained in Roswell Park Memorial Institute (RPMI) 1,640 (Sigma-Aldrich) cell culture medium supplemented with 10% fetal calf serum (PAA, Linz, Austria) and cultured at 37°C and 5% CO_2_ in a humidified tissue culture incubator. To maintain cisplatin resistance in A2780cis cells, a weekly dose of 1 µM cisplatin was applied for 72 h.

### Preparation of cytospins

To prepare cytocentrifuge specimens (cytospins), cells were seeded in 6-well plates (5 × 10^5^ cells/well), were recovered for 24 h and then exposed to 10 µM cisplatin for 6 h. Cells were detached using TrypLE™ Express, washed twice with TBS and deposited on microscope slides (Superfrost) using a Cytospin 4 cytocentrifuge (Thermo Scientific) at 350 rpm for 5 min. The microscope slides were dried at room temperature (RT), fixed with 4% paraformaldehyde (PFA) and washed with water. The slides were then incubated for 30 min at 96°C in an antigen retrieval solution and afterwards washed with water. To detect total (plasma membrane-bound and intracellular) levels of CTR1, cells on cytospins were permeabilized with TBS/0.05% Tween for 10 min. To label only plasma membrane-associated CTR1, no permeabilization was performed and cells were washed with TBS. Next, unspecific binding sites were blocked using SuperBlock blocking buffer in TBS for 30 min at RT, followed by incubation with a 1:100 diluted solution of CD16/CD32 in TBS/0.05% Tween for 10 min. The cytospins were then incubated with the ^165^Ho-labeled anti-CTR1 antibody (1:50 in 0.5% BSA and 1:100 CD16/CD32 in TBS/0.05% Tween) overnight at 4°C. Next, cytospins were washed thrice with TBS/0.05% Tween and stained with the Cell-ID Intercalator-191/193Ir (diluted to a final concentration of ∼1.25 µM) in TBS/0.05% Tween for 5 min at RT. As a final step, the slides were washed twice with TBS/0.05% Tween, washed with water and were air-dried at RT.

### MTT-based cytotoxicity assay

Cells were seeded at a density of 4 × 10^3^ cells/well in 96-well plates, were recovered for 24 h and were then exposed to cisplatin (0.5–25 µM). Following 72 h continuous drug exposure cell viability was determined by an MTT-based cell proliferation and cytotoxicity assay (EZ4U, Biomedica, Vienna, Austria) following the manufacturer’s recommendations, as published (Fronik et al., 2022). Absorbance was measured at 450 nm (at 620 nm as reference) using the multimode plate reader Tecan Infinite 200 Pro (Zurich, Switzerland). Half-maximal inhibitory concentration (IC_50_) values were derived from full dose-response curves using four parameter logistic (4 PL) regression in GraphPad Prism 8 software (La Jolla, CA, United States).

### Western blot analysis

Cells were seeded in 6-well plates (7 × 10^5^ cells/well), incubated for 24 h and then exposed to 10 µM cisplatin for 6 h. Cells were harvested and proteins were isolated as described previously (Baier et al., 2022). Polyvinylidene difluoride membranes were incubated for 18 h while shaking at 4°C in TBS/0.1% Tween/3% BSA containing the following primary antibodies: anti-CTR1 rabbit polyclonal antibody (#sc-66847, 1:500), purchased from Santa Cruz Biotechnology (Dallas, TX, United States) and anti-beta-Actin mouse monoclonal antibody (#A5441, 1:2,000), purchased from Sigma-Aldrich. The membranes were then incubated for 1 h at RT in TBS/0.1% Tween/1% BSA containing the following secondary antibodies: anti-rabbit IgG HRP-linked antibody (#7074, 1:5,000), purchased from Cell Signaling Technology (Danvers, MA, United States) or goat anti-mouse-IgG (Fc specific)-peroxidase antibody (#A0168, 1:10,000), purchased from Sigma-Aldrich.

### Laser ablation-inductively coupled plasma-time-of-flight mass spectrometry

An Iridia 193 nm laser ablation system (Teledyne Photon Machines, Bozeman, MT, United States) coupled to an *icp*TOF 2R ICP-TOFMS instrument (TOFWERK AG, Thun, Switzerland) served for all laser ablation measurements. This laser ablation system is equipped with an ultrafast ablation cell (Van Malderen et al., 2020) in the Cobalt ablation chamber and the aerosol rapid introduction system (ARIS). An Ar make-up gas flow (∼0.90 L min^−1^) was introduced through the ARIS into the optimized He carrier gas flow (0.60 L min^−1^) before entering the plasma. An optimization of the laser ablation and ICP-TOFMS settings was performed daily prior to the measurements using a NIST SRM612 glass certified reference material (National Institute for Standards and Technology, Gaithersburg, MD, United States). The settings were optimized to achieve high intensities for selected masses across the whole mass range (^26^Mg^+^, ^59^Co^+^, ^115^In^+^ and ^238^U^+^) while keeping a low oxide level (based on ^238^U^16^O^+^/^238^U^+^) (≤2%) and a laser-induced elemental fractionation (based on ^238^U^+^/^232^Th^+^) of around 1. Laser ablation was performed using a circular spot size of 2 μm, a fixed dosage of two and the line scans were overlapping in *y*-direction by 1 μm, which resulted in a pixel size of 1 µm × 1 µm. In order to achieve complete ablation of the cells and the gelatin micro-droplet standards without ablation of the glass, a fluence between 0.80 and 1.00 J cm^−2^ at a repetition rate of 200 Hz was used. The laser parameters were changed for the measurement of copper to increase the sample amount per laser shot and thereby allowed the analysis of this low abundant element. Therefore, a circular laser spot size of 4 µm (resulting pixel size of 2 µm × 2 µm) and an accordingly reduced fluence of 0.6 J cm^−2^ was used. Either the standard operation mode or the CCT mode (depending on the required sensitivity) was used for all measurements. These analyses modes balance mass resolving power, sensitivity and ion transmission across the entire measured mass range and allow the analysis of ions from m/z = 14–256. The integration and read-out rate were optimized to match the laser ablation repetition rate. Instrumental parameters for LA-ICP-TOFMS measurements using either the standard or CCT mode are summarized in Supplementary Table S1. In case the CCT mode was used, the collision/reaction cell was pressurized with a mixture of H_2_/He gas with an optimized flow rate of 4.2 ml min^−1^. The following CCT parameters were used: CCT focus: 1.5 V, CCT entry lens: −40 V, CCT mass: 250 V, CCT bias: 1.5 V, CCT exit lens: −160 V.

### Data acquisition and processing of inductively coupled plasma-time-of-flight mass spectrometry data

Data was recorded using TofPilot 2.11.6.0 (TOFWERK AG, Thun, Switzerland) and saved in the open-hierarchical data format (HDF5, www.hdfgroup.org). Post-processing of the data was performed in Tofware v3.2.2.1, a TOFWERK data analysis package which is used as an add-on on IgorPro (Wavemetric Inc., OR, United States). The data processing included following steps: 1) drift correction of the mass peak position in the spectra over time *via* time-dependent mass calibration, 2) determining the peak shape, and 3) fitting and subtracting the mass spectral base-line. All post-processing steps were saved in the HDF5 files.

### Data processing of inductively coupled plasma-time-of-flight mass spectrometry analysis

The LA-ICP-TOFMS data was further processed in HDIP-v1.6.6, a laser ablation software provided by Teledyne Photon Machines (Bozeman, MT, United States), which allowed to choose the elements of interest and to export the corresponding raw images in a Tagged Image File Format (TIFF). For cell segmentation, a stack of the raw images of ^193^Ir, visualizing the nucleus, and ^63^Cu, visualizing the cytoplasm, was produced using ImageJ. This stack image was then further processed in Cellpose (Stringer et al., 2021), a python-based deep-learning segmentation algorithm, where the actual segmentation was performed. Therefore, a small area of the stack image was loaded into Cellpose, the diameter was automatically calibrated and a first segmentation was performed using the implemented *cyto model zoo*, which is optimized for images containing a cytosol signal. To further optimize the segmentation result, the automatic segmentation was manually improved by removing or editing wrong segmented cells and adding new segments, which were not automatically found, and then a new model was trained. This procedure was repeated several times using different areas of the stack until the result was satisfactory and (almost) no cells were wrongly segmented. Then, the trained and optimized model was applied on all images of interest. Finally, the segmented cells were saved as a mask and further processed using CellProfiler, an open-source software for cell image analysis (Carpenter et al., 2006). There, the cell mask and the raw images of the isotopes of interest were loaded, and the single hot pixels of each channel were removed. In the end, the integrated intensities of every channel of interest and the area and diameter of each segmented cell, based on the segmentation mask, were exported as csv files. All further data wrangling, statistics and visualization was performed in R. For all Cu measurements a stack of ^23^Na and ^31^P was used, as those samples were not labeled with an Ir-DNA intercalator and copper was only present at low concentrations, which required an independent training of the model.

Quantification and normalization (to account for measurement uncertainties) of LA-ICP-TOFMS data was based on a multi-point calibration using gelatin-based micro-droplet standards as described by Schweikert et al. (2021). Briefly, a CellenONE X1 micro-spotter and cell arrayer (Cellenion, Lyon, France) was used to produce arrays of gelatin micro-droplets of around 400 ± 5 pL (resulting in droplet diameters of around 200 µm), containing multi-element standard solutions onto glass slides. After spotting, the droplets dry within seconds due to their small size. The gelatin droplets were quantitatively ablated by LA-ICP-TOFMS and the sum of the elemental signal intensities were extracted *via* HDIP and used for external calibration. A scheme of the whole methodology is presented in Supplementary Figure S1.

## Results

### Intracellular cisplatin accumulation is quantitatively restricted in cisplatin-resistant ovarian cancer cells

**FIGURE 1:**
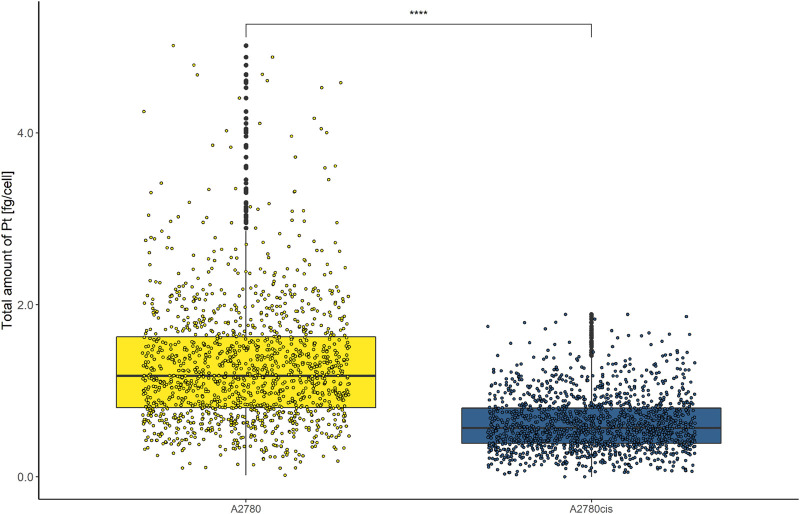
Box plots showing the concentration of Pt cell^−1^ of A2780 and A2780cis cells treated with 10 µM cisplatin for 6 h. Cytospin samples were measured by LA-ICP-TOFMS at the single-cell level. The results are based on ∼1,500 cells for each cell type. A Wilcoxon test was performed for the statistical comparison of the different cell types. Significance codes: (*) *p* ≤ 0.05, (**) *p* ≤ 0.01, (***) *p* ≤ 0.001, (****) *p* ≤ 0.0001.

Cellular cisplatin resistance was frequently associated with reduced intracellular accumulation of the drug, mainly caused by decreased drug influx and/or increased efflux (Kalayda et al., 2012; Song et al., 2022). To investigate, whether drug uptake dynamics contribute to the resistance phenotype of A2780cis at the single-cell level, A2780 and A2780cis cell lines were exposed to 10 µmol L^−1^ cisplatin for 6 h. Cytospin preparations were assessed by LA-ICP-TOFMS. The platinum amounts per single cell were obtained using gelatin micro-droplet standards (Schweikert et al., 2021). As shown in Figure 1, cisplatin accumulation was more than halved in the resistant subline as compared to the parental A2780 cell model. Average Pt concentrations of 1.28 ± 0.79 fg cell^−1^ for A2780 cells and 0.62 ± 0.34 fg cell^−1^ for A2780cis cells were assessed. The obtained values are well in agreement with the literature, where the Pt content of the same resistance model was measured by solution-based single-cell analysis (Corte Rodríguez et al., 2017).

In addition, the impact of cisplatin on the cell viability of A2780 and A2780cis was assessed by an MTT-based cytotoxicity assay (Supplementary Figure S2). Therefore, the cells were continuously exposed to different concentrations of cisplatin for 72 h and the cell viability was determined. A2780 showed a IC_50_ value of 1.56 ± 0.31 µM, while A2780cis exhibited a IC_50_ value of 6.87 ± 1.13 µM, resulting in a resistance factor of 4.4.

### Cisplatin resistance is accompanied by distinctly decreased total CTR1 levels

**FIGURE 2:**
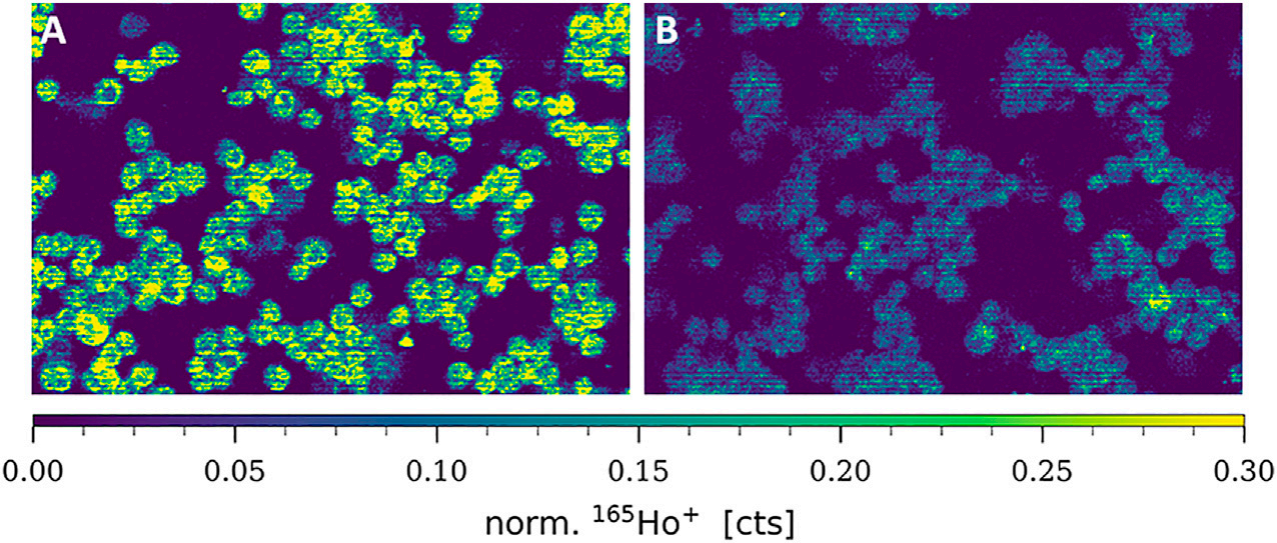
Signal intensity maps of ^165^Ho^+^, showing normalized cellular levels of total CTR1 in **(A)** A2780 and **(B)** A2780cis. Cytospin samples were measured by LA-ICP-TOFMS imaging at the single-cell level.

Cisplatin uptake is known to be based on both passive diffusion and active import *via* membrane transporters, particularly CTR1. Hence, this transporter is likewise discussed to be involved in metal-drug resistance in cancer cells and several studies previously demonstrated decreased amounts of CTR1 in resistant cancer cell models (Song et al., 2004; Zisowsky et al., 2007). In this study, total CTR1 levels (comprising plasma membrane-associated and intracellular transporter levels) were determined at the single-cell level in A2780 and A2780cis cell lines, deposited on cytospins. As can be observed in Figure 2, an ^165^Ho-tagged anti-CTR1 antibody, representing the total cellular amount of CTR1 transporter, served for imaging mass cytometry by LA-ICP-TOFMS. The signal intensity maps of ^165^Ho clearly showed, that total CTR1 levels were significantly lower in the resistant A2780cis as compared to the parental A2780 cell line. These results were additionally validated by Western blot analysis, confirming lower CTR1 levels in the resistant, as compared to the parental cell model (Supplementary Figure S3).

**FIGURE 3:**
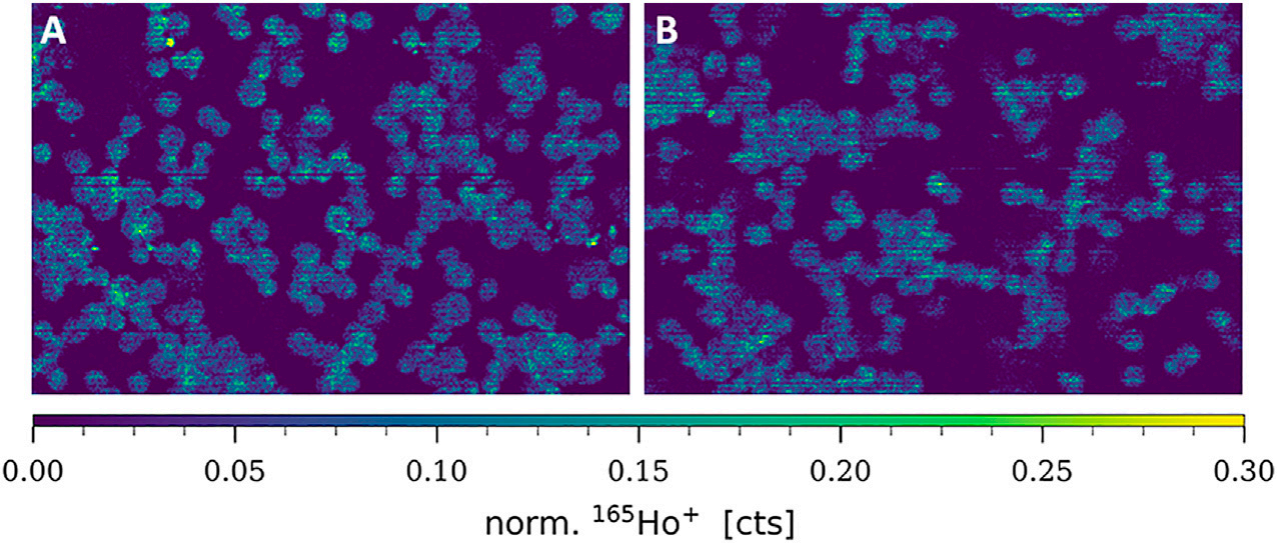
Signal intensity maps of ^165^Ho^+^, showing normalized cellular levels of plasma membrane-bound CTR1 in **(A)** A2780 and **(B)** A2780cis. Cytospin samples were measured by LA-ICP-TOFMS imaging at the single-cell level.

**FIGURE 4:**
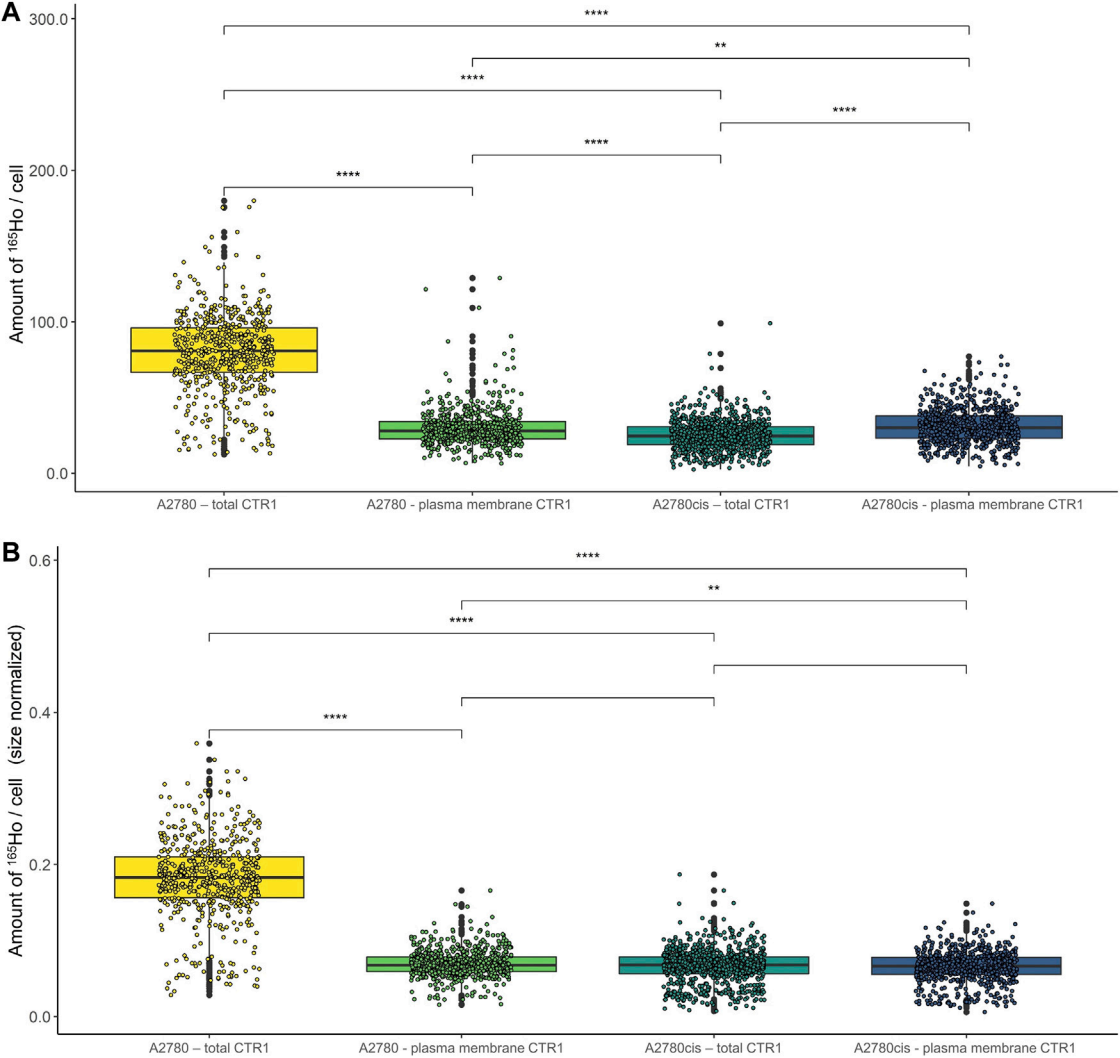
Box plots showing the relative amount of ^165^Ho cell^−1^ in A2780 and A2780cis cells. The intensity of the ^165^Ho signal corresponds to the amount of total or plasma membrane-bound CTR1. The results are shown **(A)** without and **(B)** with prior size normalization. Cytospin samples were measured by LA-ICP-TOFMS imaging at the single-cell level. The results are based on ∼1,000 to 1,500 cells for each sample. A Wilcoxon test was performed for the statistical comparison of the different cell types. Significance codes: (*) p ≤ 0.05, (**) p ≤ 0.01, (***) p ≤ 0.001, (****) p ≤ 0.0001.

CTR1 is known to be mainly located in the cellular plasma membrane, however, a significant share of intracellular CTR1 is responsible for intracellular Cu trafficking (Öhrvik and Thiele, 2014). Therefore, additional imaging mass cytometry experiments were carried out omitting the permeabilization step in order to comparatively assess the amount of total versus plasma membrane CTR1 in the sensitive (A2780) and resistant (A2780cis) ovarian cancer cell lines (Figures 3, 4). As already described above and well in accordance with the existing literature (Zisowsky et al., 2007), a distinctly and significantly lowered amount of the total copper transporter was measured in the resistant A2780cis cells, as compared to the parental A2780 cell model. The picture revealed by the plasma membrane CTR1 measurement was different, as the plasma membrane CTR1 data indicated a similarly low expression in the resistant as compared to the sensitive cell line (Figures 3, 4).

### Cisplatin sensitivity determines cisplatin-mediated impact on CTR1 expression levels

**FIGURE 5:**
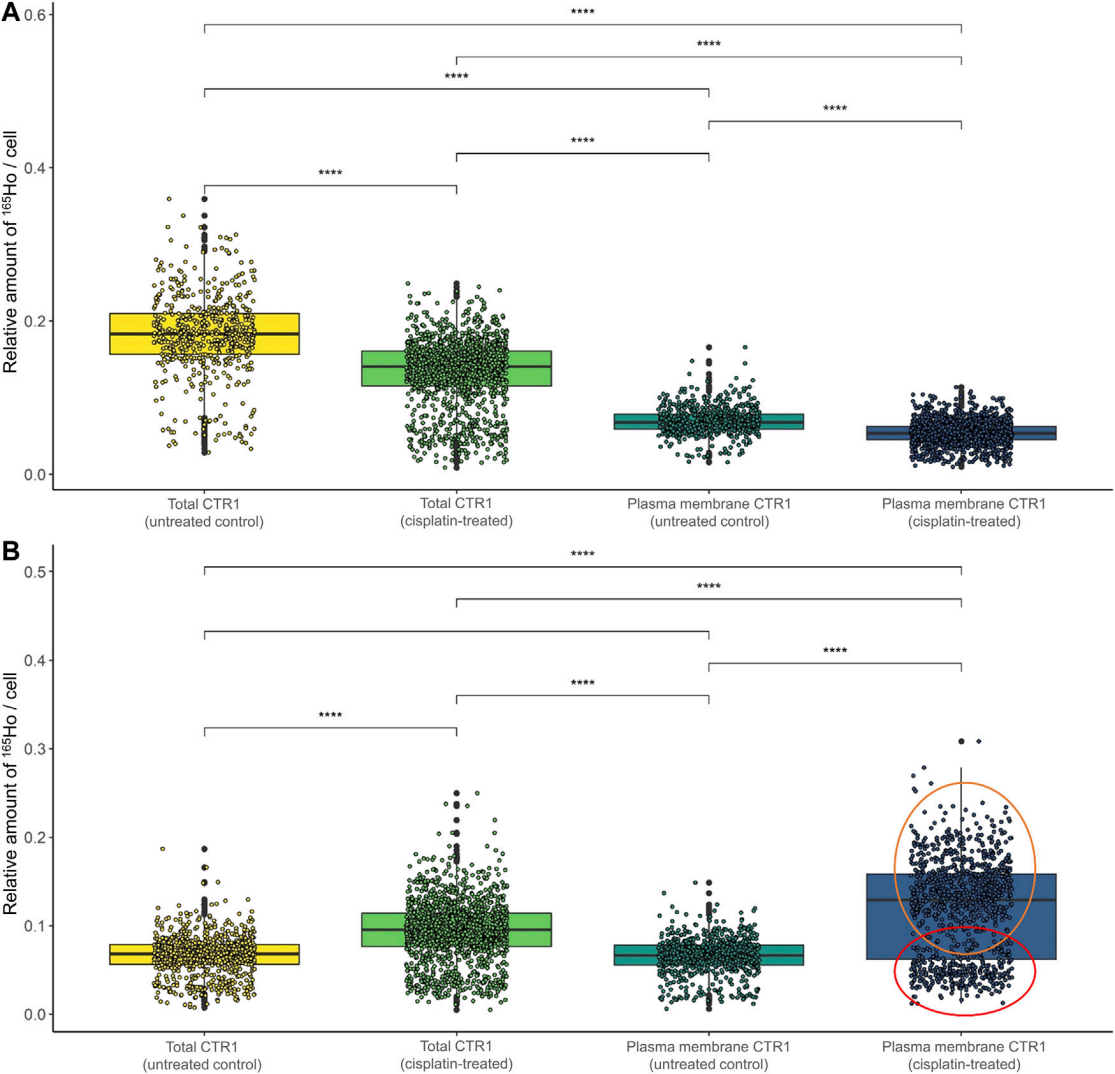
Box plots showing the relative amount of ^165^Ho cell^−1^ after size normalization in **(A)** A2780 and **(B)** A2780cis cells both untreated (control) and after treatment with 10 µM cisplatin for 6 h. The amount of ^165^Ho represents the amount of total and plasma membrane CTR1 transporter. The results are based on ∼800 to 1,500 cells for each sample. The red circle shows a cell subpopulation with a low amount of CTR1, whereas the orange circle indicates a subpopulation with a high amount. A Wilcoxon test was performed for the statistical comparison of the different cell types. Significance codes: (*) p ≤ 0.05, (**) p ≤ 0.01, (***) p ≤ 0.001, (****) p ≤ 0.0001.

Concerning the impact of cisplatin treatment on CTR1 expression levels, contradicting observations are described in the literature (Holzer, Katano, 2004; Blair et al., 2010; Kalayda et al., 2012; Schneider et al., 2017). In our study, a significant impact of the metal-drug on transporter abundance was observed (Figure 5; Supplementary Figure S4). In the parental A2780 cells, exposure to cisplatin significantly decreased total CTR1 levels (Figure 5A). Interestingly, an opposite trend was observed in the A2780cis cells, where cisplatin induced a significant upregulation of the CTR1 transporter (Figure 5B), both at the level of total and plasma membrane CTR1. In the sensitive model the apparent decrease upon drug exposure concerned primarily the total CTR1, indicating that intracellular CTR1 was affected foremost by the chemotherapeutic application. The high affinity copper transporter CTR1 exists as a full-length protein with varying degrees of glycosylation and as a lower-molecular weight, truncated form (tCTR1), lacking the metal-binding extracellular domain. The histidine- and methionine-rich metal-binding ectodomain facilitates Cu import and influx of platinum-based drugs, such as cisplatin requires the methionine extracellular domain. Cathepsin L/B endolysosomal proteases-induced cleavage of the ectodomain leads to a reduction of the import activity of Cu by 50% as compared to the full-length CTR1 and depends on the structurally related CTR2. Truncated CTR1 is required for trafficking of endosomal Cu (Öhrvik et al., 2013; Öhrvik et al., 2016). Bulk experiments determining CTR1 levels by Western blot indicated an impact of the metal drug primarily in the resistant A2780cis cells, cisplatin treatment tended to slightly decrease the glycosylated full-length CTR1, but at the same time induced a distinct increase of the lower-molecular weight protein band, suggesting an impact of the drug on the truncated CTR1 in the resistant cell line (Supplementary Figure S3).

Surprisingly, in the resistance model after cisplatin treatment, a share of single cells showed a very high expression of plasma membrane CTR1 upon drug treatment (Supplementary Figure S4D). A bimodal distribution was obtained in boxplots following cell size normalization, which can be easily performed in laser ablation ICP-MS imaging and can be used to account for cell size effects (Figure 5B).

### Intracellular cisplatin accumulation correlates with CTR1 transporter levels

**FIGURE 6:**
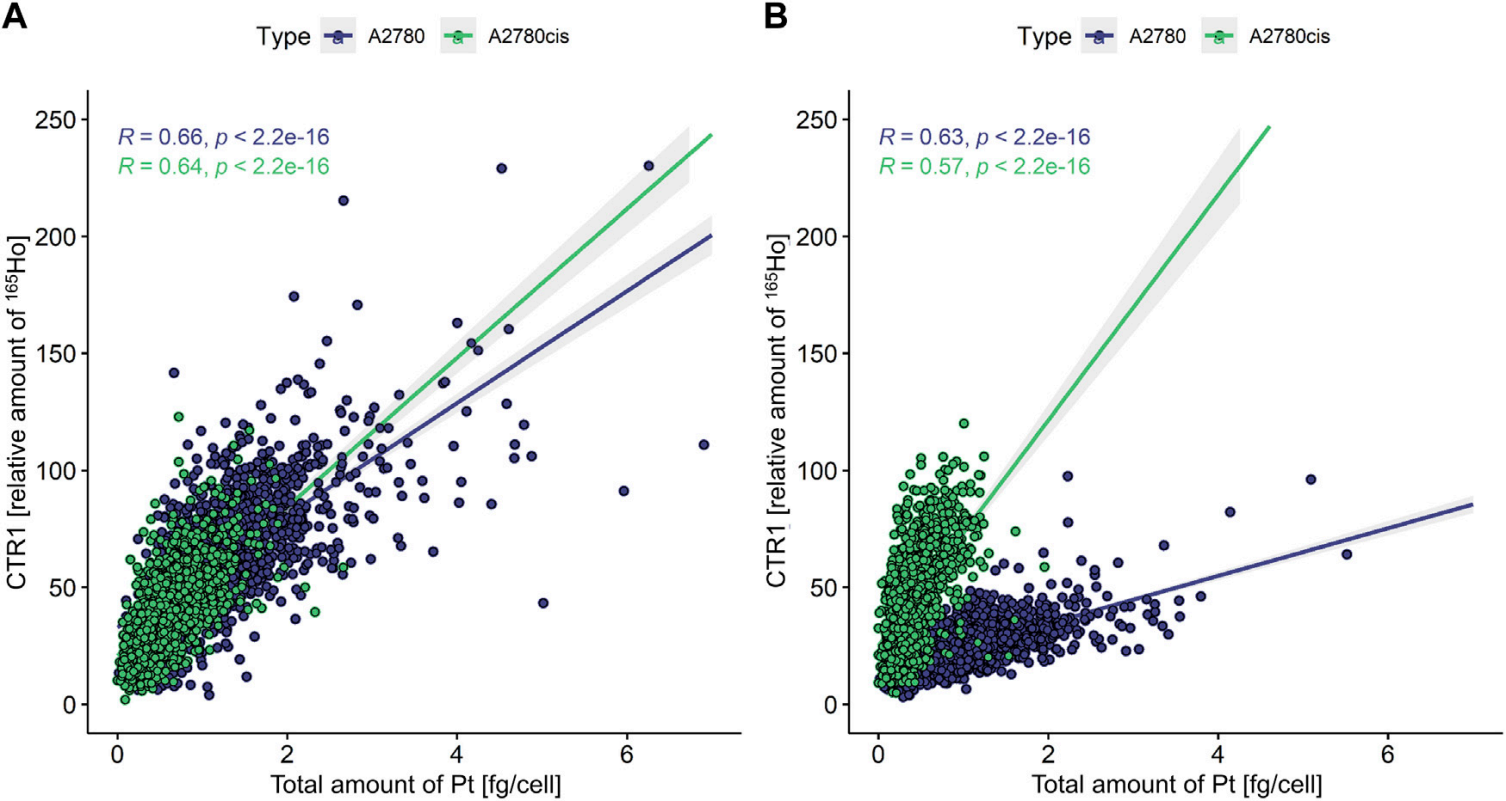
Scatterplot showing the total amount of Pt [fg cell^−1^] versus the relative amount of ^165^Ho [counts cell^−1^] representing the amount of **(A)** total and **(B)** plasma membrane-associated CTR1. A Pearson correlation was calculated between those two variables for both A2780 and A2780cis cells. A value of 0.00–0.10 indicates a negligible correlation, 0.10–0.39 a weak correlation, 0.40–0.69 a moderate correlation, 0.70–0.89 a strong correlation, and a value of 0.90–1.00 a very strong correlation (Schober et al., 2018).

Finally, intracellular cisplatin accumulation was investigated along with total or plasma membrane CTR1 expression levels (Figure 6). A Pearson correlation coefficient was calculated between intracellular Pt concentration and CTR1 levels, with values of 0.00–0.10 indicating a negligible correlation, 0.10–0.39 a weak correlation, 0.40–0.69 a moderate correlation, 0.70–0.89 a strong correlation, and 0.90–1.00 a very strong correlation (Schober et al., 2018). Here, moderate correlations were observed between total as well as plasma membrane CTR1 and Pt concentrations (Figure 6A) for both A2780 and A2780cis cells. Interestingly, regarding plasma membrane-associated CTR1, the resistant cell line showed high levels of CTR1, but low Pt concentrations, whereas the opposite pattern was observed for the parental cells (Figure 6B).

### Cisplatin resistance is associated with a distinctly decreased intracellular Cu abundance

**FIGURE 7:**
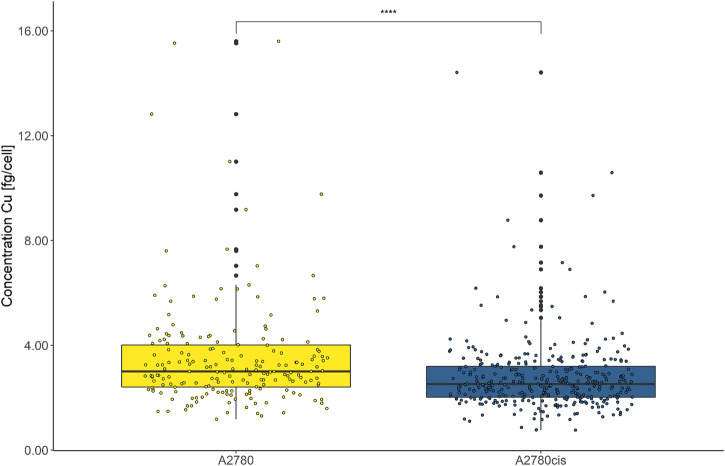
Box plots showing the concentration of intracellular Cu cell^−1^ of A2780 and A2780cis cells. Cytospin samples were measured by LA-ICP-TOFMS at the single-cell level. All values below the LOD were removed. A Wilcoxon test was performed for the statistical comparison of the different cell types. Significance codes: (*) p ≤ 0.05, (**) p ≤ 0.01, (***) p ≤ 0.001, (****) p ≤ 0.0001.

Intracellular Cu levels were investigated as CTR1 is key for cellular Cu homeostasis and several studies found a relation between intracellular Cu and cancer disease progression (Chen et al., 2020). Both cell lines were assessed by LA-ICP-TOFMS without immunohistochemistry preparation, reducing sample preparation steps to a minimum. This was a necessity, since Cu contaminations in the labeling buffers would otherwise result in biased intracellular Cu accumulation data, impeding the assessment of biological-relevant Cu concentrations. As depicted in Figure 7, the overall cellular Cu content was remarkably low and from ∼2000 segmented and analyzed cells only ∼200 cells of each cell line showed values above the limit of detection (LOD) of this method. Intracellular Cu levels of the remaining, decipherable cells above the LOD indicated significantly decreased Cu abundance in the resistant A2780cis compared to the parental A2780 cells.

Additionally, we analyzed the impact of cisplatin on intracellular Cu levels and found a distinct decrease in the Cu content in cisplatin-exposed vs. untreated cells, however, Cu levels of all treated cells were below the LOD, making the exact assessment of the treatment impact on intracellular Cu impossible.

## Discussion

For the first time, cutting edge bioimaging was applied to study cisplatin accumulation along with CTR1 expression and its crosstalk to physiological Cu homeostasis at the single-cell level. It has been more than 20 years since active transport of cisplatin by CTR1 was hypothesized. Ever since, the involvement of CTR1 in cisplatin accumulation and its effect on intra/extracellular Cu trafficking was controversially discussed, especially based on the observation of reduced cisplatin accumulation in cell models featuring an acquired resistance phenotype. There are indications that a reduction of CTR1 in resistant cancer cells is, among others, responsible for the decreased drug uptake (Ishida et al., 2002; Song et al., 2004; Zisowsky et al., 2007). However, the molecular details of this resistance mechanism are still not fully understood and further in-depth studies are needed. The here applied single-cell method was designed for *in vitro* experiments. It relies on spatially highly resolved laser ablation ICP-TOFMS imaging applied to cytospin-prepared cells. Absolute quantification of intracellular Pt and Cu concentrations is enabled by performing calibrations prior to analysis. Compared to suspension-based elemental single-cell methods, the workflow has the advantage of both visualizing the cells and allowing further data evaluation and statistical analyses after extracting single-cell information from images *via* segmentation. Additionally, cell integrity and cell size are easily accessible parameters when using cytospins (Schoeberl et al., 2021).

Cisplatin-sensitive A2780 ovarian cancer cells and its cisplatin-resistant derivative A2780cis served as an ideal cell model to study platinum accumulation and Cu homeostasis together with CTR1 transporter protein expression in the context of acquired resistance. As already shown in previous bulk analyses (Corte Rodríguez et al., 2017), the A2780cis cells were characterized by a decreased platinum concentration, with Pt content per cell twice as high in the sensitive as compared to the resistant cell line (compare Figure 1).

Concerning total CTR1 expression determined in our study by imaging mass cytometry, previous bulk method-based observations on reduced CTR1 expression levels accompanying acquired cisplatin resistance were confirmed (Zisowsky et al., 2007). We observed total CTR1 levels to be clearly reduced in A2780cis as compared to the parental A2780 cells (compare Figure 2). Regarding cell surface CTR1, parental A2780 cells exhibited distinctly lowered amounts of CTR1, as compared to total CTR1, indicating a preferential localization of CTR1 at intracellular sites. In contrast, A2780cis cells displayed plasma membrane CTR1 to be on a similar low level as total CTR1, implying that the Cu transporter is primarily located at the plasma membrane in the cisplatin-resistant cell line (compare Figures 3, 4). Under a normal physiological copper homeostasis, CTR1 is described to be mainly located at the plasma membrane. However, when the cells are medicated with a high amount of Cu, CTR1 is internalized to Early Endosomal Antigen (EEA1)- and Rab5-marked compartments by endocytosis. After removal of extracellular Cu, the transport protein recycles back to the plasma membrane *via* the slower recycling endosomes (Mandal et al., 2020). Öhrvik and Thiele (2014) suggested that the transport of Cu occurred *via* passive transport, driven by a concentration gradient or through other mechanisms, which stimulate Cu to pass through the CTR1 pore. Regarding platinum-based drugs, the authors proposed, that platinum uptake occurs *via* endocytosis instead of passage through the CTR1 pore (Öhrvik and Thiele, 2014).

The impact of cisplatin treatment on CTR1 expression is a matter of ongoing debate and contradicting observations were reported (Holzer and Katano, 2004; Blair et al., 2010; Kalayda et al., 2012; Schneider et al., 2017). While Blair et al. (2010) and Holzer et al. (2006) described a degradation of CTR1 after exposure to cisplatin, the group of Kalayda et al. (2012) could not see any loss of CTR1 through treatment. Schneider et al. (2017) described a varying effect on sensitive and resistant cells (A2780 and A2780cis, respectively), however, a combination of cisplatin and sorafenib was applied in this study. Our investigations demonstrated different impacts of cisplatin on CTR1 expression on the single-cell level according to the resistance status. In the sensitive A2780 cell line, a moderate decrease of both total and cell surface CTR1 levels was observed after cisplatin. Surprisingly, total and especially plasma membrane CTR1 distinctly increased following cisplatin treatment of A2780cis cells (compare Figure 5 and Supplementary Figure S4). These unexpected results were confirmed by Western blot analyses, exhibiting a markedly increase of the lower molecular weight variant of CTR1 in A2780cis cell extracts, while the high-molecular weight isoform decreased (compare Supplementary Figure S3).

In addition, the correlation of intracellular Pt content and plasma membrane-bound CTR1 yielded two highly varying regression lines in case of A2780cis, but not the parental cell line (compare Figure 6). This suggests that CTR1 is not only downregulated in the resistant cell line, but that A2780cis cells upregulate a CTR1 variant in response to cisplatin, which preferentially localizes to the cell membrane, but lacks efficient cisplatin transporting capacity. The high affinity copper uptake protein CTR1 is represented by a full-length protein with varying and tissue-specific degrees of glycosylation, as well as a truncated version of CTR1 (tCTR1), lacking the metal-binding extracellular domain. Formation of tCTR1 depends on the structurally related CTR2 and is mediated by Cathepsin L/B endolysosomal proteases-induced cleavage of the CTR1 ectodomain. This proteolytic processing leads to a reduction of the import activity of Cu by 50% as compared to the full-length CTR1 (Öhrvik et al., 2013; Öhrvik et al., 2016). Interestingly, high CTR2/CTR1 ratios were described to correlate with poorer chemotherapy response and prognosis (Yoshida et al., 2013; Öhrvik and Thiele, 2015, p. 1). Whether, the low-molecular weight variant upregulated in response to cisplatin specifically in A2780cis cells in our immunoblot analysis is identical with the truncated CTR1 version, lacking the metal-binding ectodomain, needs to be determined in further investigations.

Despite the overall rather low Cu concentrations in the investigated ovarian cancer cell lines, an even lower accumulation was found in the resistant compared to the sensitive cell model, reflecting reduced CTR1 uptake transporter (compare Figure 7). The direct exposure to cisplatin led to a marked decrease in copper accumulation to undetectable levels in both cell lines. This effect was already discussed in the literature (Ishida et al., 2002) and suggests competition between copper and cisplatin as a substrate for CTR1-mediated cellular uptake. Our study showed this interrelation for the first time at the single-cell level.

In conclusion, it could be shown in this study that the resistant ovarian cancer cells as compared to the sensitive cells are characterized by a decreased platinum accumulation, a decreased concentration of copper, lower levels of total CTR1 and a high correlation between total CTR1 and Pt. Interestingly, the amount of cell surface and total transporter and its relation were shown to be different in A2780 and A2780cis cells and two distinct subpopulations in response to cisplatin were observed in the resistant cell line when visualizing the plasma membrane transporter. Opposite effects on the amount of CTR1 cell surface expression in response to cisplatin were observed according to the resistance phenotype, indicating a possible role of a cisplatin transport-deficient truncated CTR1 variant in acquired cisplatin resistance. Moreover, we confirmed a competitive impact of cisplatin treatment on the Cu homeostasis on a single-cell level.

Summarizing, the presented single-cell LA-ICP-TOFMS imaging method did not only allow to confirm previous results concerning the interplay of CTR1 expression, Cu homeostasis and cisplatin response, but also delivered unexpected novel information with regard to the dynamic regulation of subcellular localization and membrane exposure of this important cisplatin resistance transporter.

## Data Availability

The raw data supporting the conclusion of this article will be made available by the authors, without undue reservation.
