# Supplementary material for: The copper transporter CTR1 and cisplatin accumulation at the single-cell level by LA-ICP-TOFMS
Source: Front Mol Biosci. 2022 Nov 28;9:1055356. doi: 10.3389/fmolb.2022.1055356 (PMC9742377; doi:10.3389/fmolb.2022.1055356)
Supplement: Supplementary file 1 [file DataSheet1.pdf]

Supplementary Material

1 Supplementary tables

Supplementary Table S1. Instrumental parameters for LA-ICP-TOFMS measurements.

	Standard mode	H_2/He gas mode
RF Power [W]	1440	1440
Sampling depth [mm]	3.5	3.4
Cone materials	Ni	Ni
Plasma gas flow [L min^{-1}]	14	14
Auxiliary gas flow [L min^{-1}]	0.80	0.80
Nebulizer gas flow [L min^{-1}]	1.03	1.03
CCT1 flow rate [mL min-1]	-	4.2
Sample introduction system	Aerosol rapid introduction system (ARIS)	Aerosol rapid introduction system (ARIS)
m/z range	14-256	14-256

2 Supplementary Figures

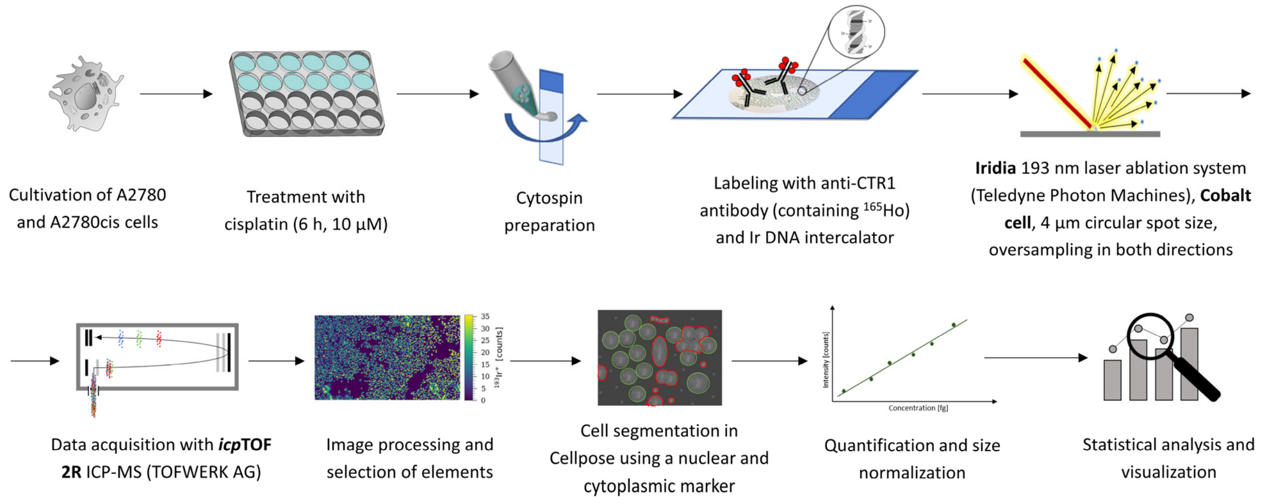

Supplementary Figure S1. Scheme of the methodology.

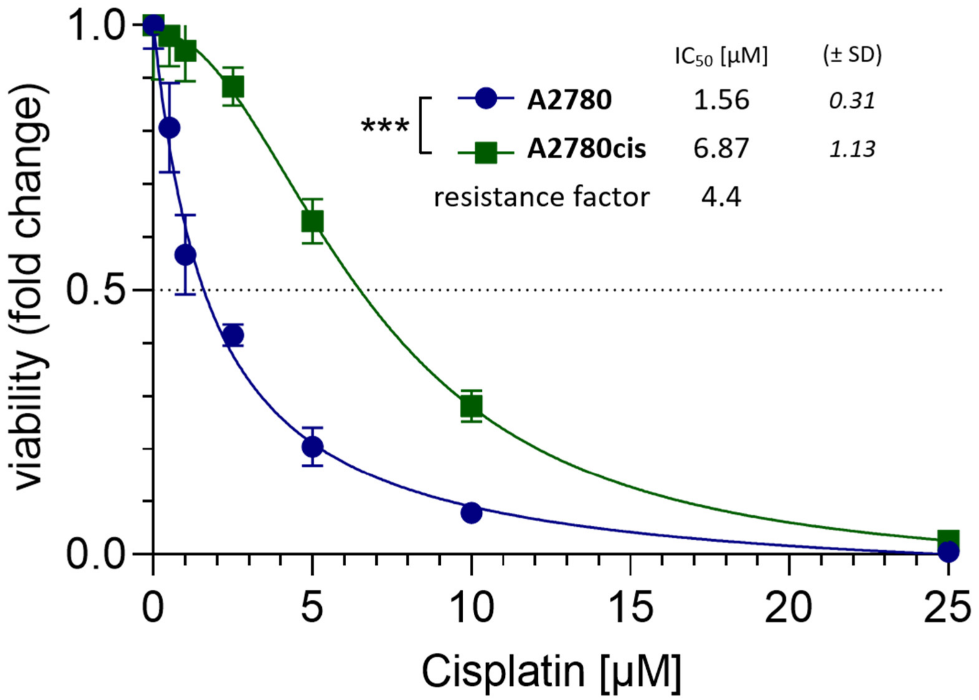

Supplementary Figure S2. Impact of cisplatin on cell viability of A2780 and A2780cis cells. Cells were continuously exposed to the indicated concentrations of cisplatin for 72 h and cell viability was determined using an MTT-based cytotoxicity assay. Data points are depicted as mean ± SD of four independent experiments. A two-way ANOVA was performed for statistical analysis. IC_{50} values were derived from dose-response curves using the four parameter non-linear regression model. Resistance factor was calculated by dividing the mean IC_{50} value of resistant A2780cis by the one of parental A2780 cells.

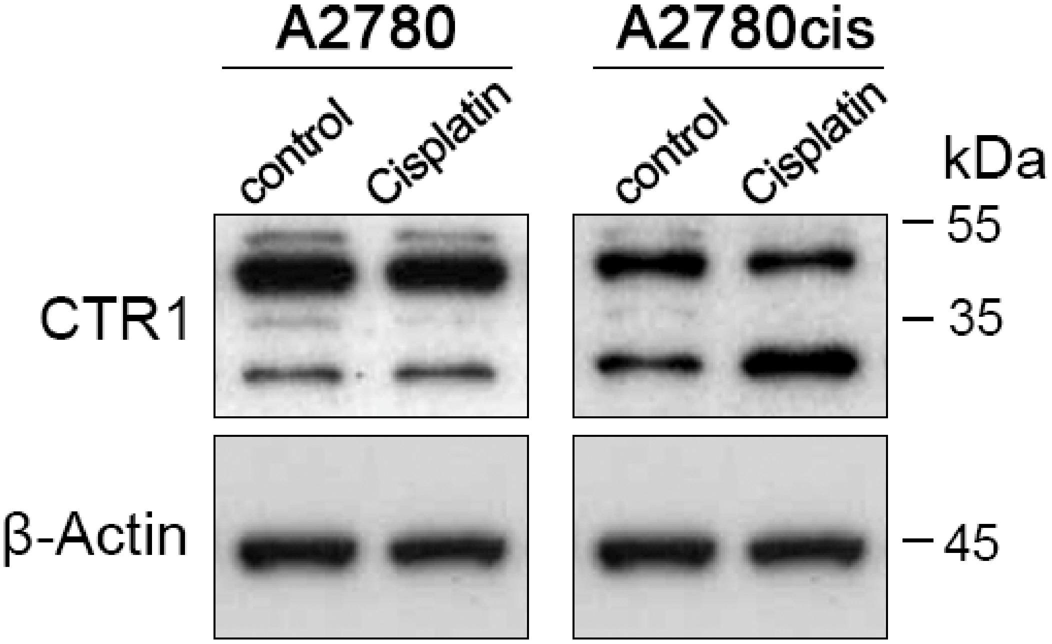

Supplementary Figure S3. Western blot of total CTR1 in A2780 and A2780cis with and without prior exposure to cisplatin for 6 h. The bands indicate high-molecular weight mature CTR1, non-glycosylated precursor CTR1 and lower-molecular weight truncated CTR1.

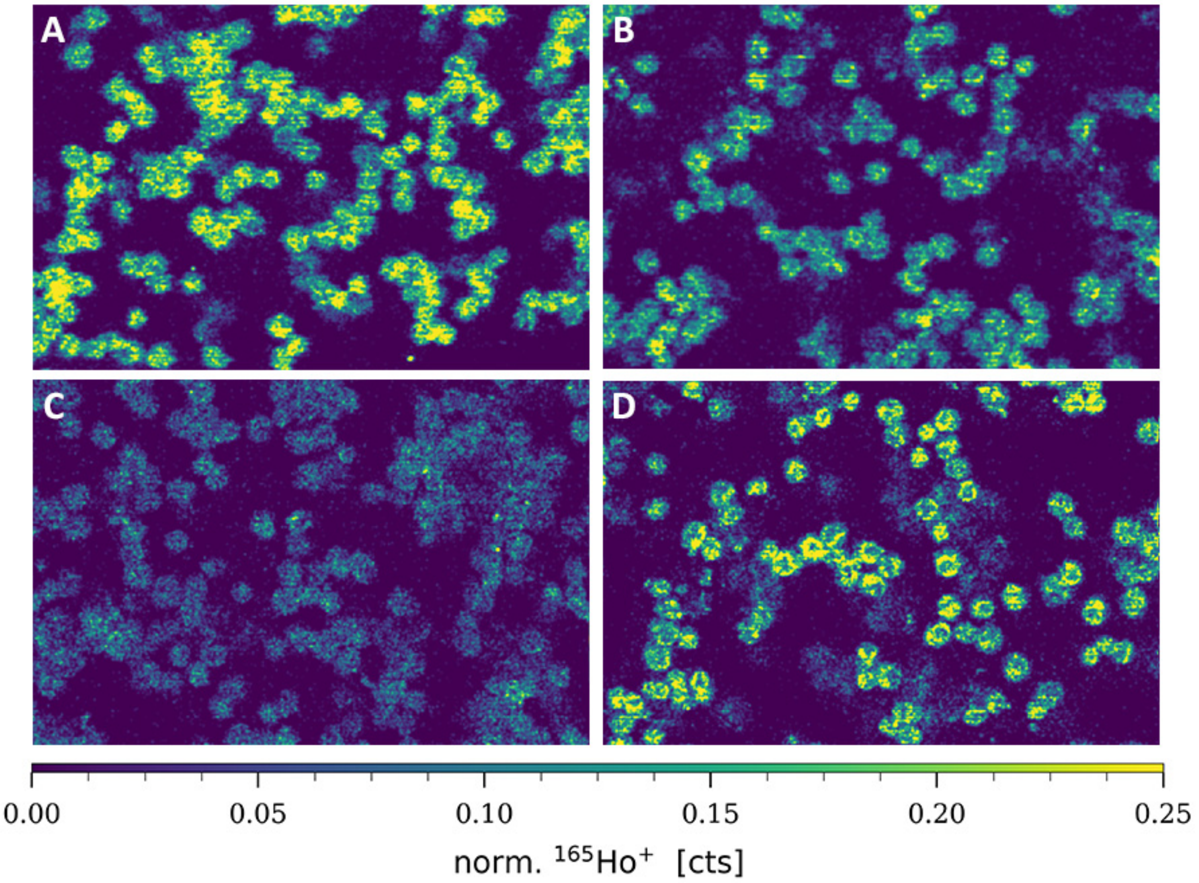

Supplementary Figure S4. Signal intensity maps of ^{165}Ho cell^{-1}, showing normalized total CTR1 levels in (A) A2780 and (B) A2780cis or plasma membrane CTR1 levels in (C) A2780 and (D) A2780cis following treatment with 10 µM cisplatin for 6 h. Cytospin samples were measured by LA-ICP-TOFMS imaging at the single-cell level.
